# Mutations of *PIK3CA *in gastric adenocarcinoma

**DOI:** 10.1186/1471-2407-5-29

**Published:** 2005-03-23

**Authors:** Vivian Sze Wing Li, Chi Wai Wong, Tsun Leung Chan, Agnes Sze Wah Chan, Wei Zhao, Kent-Man Chu, Samuel So, Xin Chen, Siu Tsan Yuen, Suet Yi Leung

**Affiliations:** 1 Department of Pathology, The University of Hong Kong, Queen Mary Hospital, Hong Kong; 2 Department of Surgery, The University of Hong Kong, Queen Mary Hospital, Hong Kong; 3 Department of Surgery, Stanford University School of Medicine, Stanford, CA 94305, USA; 4 Department of Biopharmaceutical Sciences, University of California, San Francisco, USA

## Abstract

**Background:**

Activation of the phosphatidylinositol 3-kinase (PI3K) through mutational inactivation of *PTEN *tumour suppressor gene is common in diverse cancer types, but rarely reported in gastric cancer. Recently, mutations in *PIK3CA*, which encodes the p110α catalytic subunit of PI3K, have been identified in various human cancers, including 3 of 12 gastric cancers. Eighty percent of these reported mutations clustered within 2 regions involving the helical and kinase domains. *In vitro *study on one of the "hot-spot" mutants has demonstrated it as an activating mutation.

**Methods:**

Based on these data, we initiated *PIK3CA *mutation screening in 94 human gastric cancers by direct sequencing of the gene regions in which 80% of all the known *PIK3CA *mutations were found. We also examined *PIK3CA *expression level by extracting data from the previous large-scale gene expression profiling study. Using Significance Analysis of Microarrays (SAM), we further searched for genes that show correlating expression with *PIK3CA*.

**Results:**

We have identified *PIK3CA *mutations in 4 cases (4.3%), all involving the previously reported hotspots. Among these 4 cases, 3 tumours demonstrated microsatellite instability and 2 tumours harboured concurrent *KRAS *mutation. Data extracted from microarray studies showed an increased expression of *PIK3CA *in gastric cancers when compared with the non-neoplastic gastric mucosae (*p *< 0.001). SAM further identified 2910 genes whose expression levels were positively associated with that of *PIK3CA*.

**Conclusion:**

Our data suggested that activation of the PI3K signalling pathway in gastric cancer may be achieved through up-regulation or mutation of *PIK3CA*, in which the latter may be a consequence of mismatch repair deficiency.

## Background

The phosphatidylinositol 3-kinase (PI3K)-AKT signalling pathway is involved in the regulation of diverse cellular processes, including cell growth, survival and motility. Abnormal activation of this pathway is frequently observed in various cancer types, leading to aberrant cell cycle progression, altered adhesion and motility, inhibition of apoptosis and induction of angiogenesis [1]. It has been previously reported that genetic alterations involving various members along this signalling pathway could lead to its activation in cancer. These include mutation, allelic loss or promoter methylation of the negative regulator *PTEN *[2]; or alternatively, chromosomal amplification or over-expression of the positive regulators *PIK3CA *[3-5] and the various *AKT *kinases [6,7]. Furthermore, changes in other related pathways that are commonly altered in cancer, such as those involved in growth factor stimulation via the G-protein-coupled receptors or through direct interaction with the activated form of small GTPase RAS, can also lead to PI3K-AKT pathway activation [8]. Activation of this pathway results in the phosphorylation of AKT at Thr-308/309 and Ser-473/474. These phosphorylated forms of AKT proteins have been detected by Western blot or immunohistochemistry in various cancer types, suggesting the frequent activation of PI3K-AKT pathway in the carcinogenic process [7,9].

Although genetic changes along the PI3K-AKT pathway have been repeatedly documented in brain, ovarian, endometrial, breast, prostate and thyroid cancers [1,2], reports on its mechanism of activation in gastric cancer are limited. Gastric cancer is the second most common cancer worldwide but its molecular basis of tumourigenesis is still poorly understood. Previous immunohistochemical study has demonstrated the presence of the phosphorylated form of AKT in 78% of gastric cancer [10], suggesting that activation of this pathway may also be common in gastric cancer. Though loss of heterozygosity (LOH) involving the *PTEN *locus has been demonstrated in 47% of gastric cancer in a recent study, mutation or promoter methylation was absent even in cases with LOH [11]. Thus data from this study could not support the two-hit inactivation of *PTEN *in gastric cancer, while the biological significance of *PTEN *haploinsufficiency remains controversial. Alternatively, amplification of *AKT1 *has been reported in a single case of gastric cancer [12], and amplification of *PIK3CA *associated with elevated mRNA levels has been found in 36% of gastric cancer [11]. More recently, Samuels *et al. *screened a diverse spectrum of human cancers for mutation in 16 *PI3K *or *PI3K*-like genes and found a high frequency of somatic mutation in *PIK3CA*, which encodes the p110α catalytic subunit. Major screening in colorectal cancer (CRC) identified *PIK3CA *mutations in 74 out of 234 (32%) cases, while mutations were also noted in 3 out of 12 (25%) gastric cancers. Reported mutations were mostly of missense type, and clustered within 2 regions in the helical and kinase domains. Expression of a "hot-spot" mutant, H1047R, conferred a significant up-regulation of lipid kinase activity of PIK3CA, suggesting it as an activating mutation [13]. In this study, we have examined a series of 94 human gastric adenocarcinomas for *PIK3CA *mutation. We have also examined *PIK3CA *expression level by extracting data from a large-scale gene expression profiling study previously performed for these cases [14,15]. Using SAM, genes with significant correlating expression with *PIK3CA *have also been identified.

## Methods

### Patient samples preparation

DNA samples used for sequencing were prepared from frozen tumour and non-tumour gastric mucosae from 94 gastric cancer patients who underwent gastrectomy in the Department of Surgery, Queen Mary Hospital, The University of Hong Kong, as previously described [16]. Majority of the frozen samples (n = 81) showed tumour component of over 70%, whereas in 13 cases a lower proportion between 50 to 70% was accepted due to the tumours' inherent diffuse infiltrative nature with entrapment of non-neoplastic components. Analysis for microsatellite instability (MSI), *BRAF *and *KRAS *mutation have been performed and reported previously [16]. RNA preparation and gene expression profiling using a cDNA microarray containing 44,500 cDNA clones, representing around 30,300 unique genes, has been performed and reported in 90 of these tumours in comparison to 22 non-tumour gastric mucosae [14,15]. This study was approved by the Ethics Committee of the University of Hong Kong.

### Mutational screening

Mutation screening of *PIK3CA *was performed for exons 9 and 20, covering the mutational hotspots; and for exon 18, from which a mutation was found in a gastric cancer. Mutations in these 3 exons constituted 80% of all *PIK3CA *mutations detected in the previous study [13]. *PIK3CA *intron-specific external amplification primers and internal sequencing primers were designed according to the previous study [13] with some modifications [see Additional file 1]. In particular, primers for exon 9 have been modified to avoid amplification of homologous sequences located in other chromosomes. PCR products were generated using the external primers and directly sequenced using the internal primers with the DYEnamic™ ET Terminator Cycle Sequencing Kit (Amersham Biosciences, Freiburg, Germany) according to the manufacturer's instruction. Electrophoresis was performed in the ABI Prism^® ^3700 DNA Analyzer (Applied Biosystems, Foster City, CA, USA). For each exon, PCR products were generated from 2 independent PCR reactions for sequencing of the forward and reverse strands. For exon 9, 2 independent PCR followed by sequencing of the forward strand were performed. Analysis of the chromatograms was performed using the mutation analysis software Mutation *Explorer*™ (SoftGenetics, State College, PA, USA).

### Extraction of expression data and statistical analysis

Gene expression data were extracted from the microarray database containing 126 samples (90 gastric cancers, 14 lymph node metastasis and 22 non-tumour gastric mucosae) based on a 3-fold signal above background ratio for either channel and with 80% good data [14]. Gene expression data from 20,336 cDNA clones satisfied this selection criteria and were extracted, which included a cDNA clone corresponding to *PIK3CA *(IMAGE clone number 345430, GenBank accession no. W72473). Expression data for *PIK3CA *was extracted and the differences in expression levels between tumour and non-tumour tissues were examined using the Student's *t*-Test. SAM was performed to identify genes with significant correlating expression with *PIK3CA *[17]. The missing values in the dataset were estimated by a K-nearest neighbours impute algorithm using 10 nearest neighbour [18] followed by 5000 permutations in the SAM analysis.

## Results

**Figure 1:**
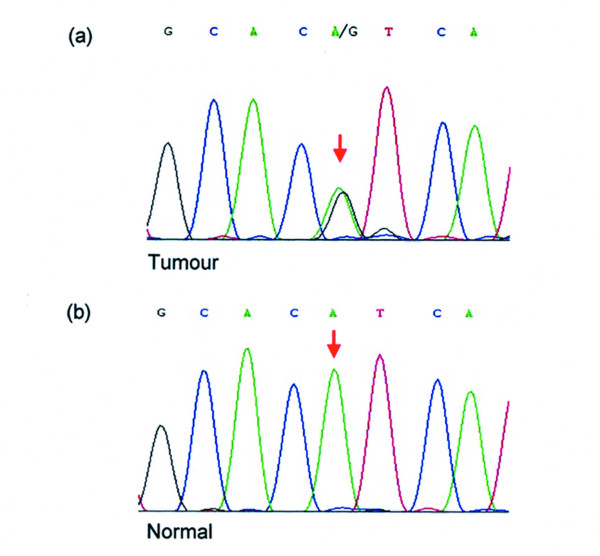
**Representative sequence chromatogram. **(a) Sequence chromatogram from a case of gastric cancer with H1047R (A3140G) mutation within exon 20 of *PIK3CA*. Site of mutation is denoted by the red arrow. (b) Sequence chromatogram of the corresponding normal mucosa from the same case showing the absence of mutation (red arrow), and thus confirming the somatic nature of the mutation.

Among the 94 gastric adenocarcinoma analysed, we have detected *PIK3CA *mutation in 4 cases. Two cases harboured the mutation A3140G (H1047R) in exon 20, and the other 2 cases with mutations G1624A (E542K) and G1633A (E545K) in exon 9. Representative sequence chromatograms are shown in figure 1. All four mutations were absent in the corresponding non-neoplastic mucosae and thus were confirmed as somatic mutations. Though the overall mutation frequency (4.3%) was lower than that of the previous study, the nature of the 4 mutations found were consistent with those identified at the reported hotspots. In particular, the H1047R mutation has been reported in 2 gastric cancers and 15 colorectal cancers [13]. While the E542K and the E545K mutations were not found in gastric cancer in the previous series, a large number of colorectal tumours did harbour these 2 mutations.

*PIK3CA *mutation spectrum and their corresponding clinico-pathological features were listed in Table 1. We noted a higher tendency of high-level MSI in gastric cancers with *PIK3CA *mutations (3 in 4, 75%) than in those without (18 in 90, 20%). Moreover, though the overall incidence of *KRAS *mutation in the studied population was low (8 in 94), 2 of the 4 gastric cancers with *PIK3CA *mutation also harboured a *KRAS *mutation.

Since over-expression of *PIK3CA *has been reported in gastric cancer [11], we have also extracted *PIK3CA *expression data from our previous cDNA microarray study of these cases [14,15]. We have confirmed that expression level of *PIK3CA *was significantly higher in gastric cancers (n = 87, mean = 0.099, SD = 0.428) when compared with non-neoplastic gastric mucosae (n = 22, mean = -0.418, SD = 0.426; Student's *t*-Test, *p *< 0.001). Using *PIK3CA *expression level as a continuous variable for SAM analysis [17], we found 2910 cDNA clones (corresponding to about 2546 unique genes) whose expression associated positively with *PIK3CA *expression (median number of false significant = 0.372, Delta = 1.107) [see Additional file 2]. Interestingly, no gene was found to be negatively associated with *PIK3CA *expression.

## Discussion

In this study, we have reported the presence of *PIK3CA *gene mutation in 4.3% of gastric cancer. A high tendency (3 in 4) of mismatch repair deficiency was noted in cases harbouring *PIK3CA *mutation. Though the small number of *PIK3CA *mutations in our study may not justify statistical claim of significance; suggestion of such, despite of its not being mentioned by the authors, can be found from a previous study in CRC by Samuels *et al.*. From their study of 33 MSI and 201 microsatellite stable (MSS) CRC cases, *PIK3CA *mutation was present in 48% of the MSI tumours, but only in 29% of the MSS tumours. A significant association would have been revealed if statistical analysis had been applied (Fisher's exact test, *p *= 0.014) [13]. Gastrointestinal tract cancers with MSI are known to have a different molecular pathway of tumour evolution compared with their MSS counterparts [19,20]. This can be attributed to their propensity for frameshift mutations in repeat sequences, resulting in selective disruption of genes with such sequences within their coding regions. With 2 poly-adenine tracts within its coding region, *PTEN *can be inactivated through frameshift mutations in MSI CRC, resulting in the selective targeting of the PI3K-AKT signalling pathway [21,22]. It is also known that mismatch repair deficiency would lead to an elevated rate of missense mutation due to impaired single nucleotide mismatch repair [23]. Thus, the observed higher incidence of *PIK3CA *missense mutation in MSI colorectal and gastric cancers suggests yet another mechanism for the activation of the PI3K-AKT signalling pathway through mismatch repair deficiency.

Our data also showed a higher tendency of *KRAS *mutation in cases with *PIK3CA *mutations (2 in 4) than in those without (6 in 90). Yet again due to the low incidence of both mutations in our samples, statistical significance may not be claimed. In the study by Samuels *et al.*, some of the colorectal tumours with *PIK3CA *mutation also harboured *KRAS *or *BRAF *mutation [13]. The PI3K-AKT pathway is known to have a close association with the RAS-MEKK signalling pathway [8]. Constitutively active RAS can interact with the catalytic subunit of PI3K and lead to its activation. Ras-dependent PI3K activation contributes to the transforming phenotype by mediating anchorage-independent growth, cytoskeletal reorganisation and apoptosis evasion. It has been observed that genes involved in the same signalling pathway may manifest mutations in cancer cells in a mutually exclusive manner, presumably due to the lack of selective growth advantage in having a second hit in the already altered pathway. A prominent example is the mutually exclusive occurrence of the *BRAF *hotspot mutation (V600E) and *KRAS *mutations in colorectal cancer [24,25]. However, there exist other examples of alterations in multiple components of the same signalling pathway that may lead to a multi-level modulation of its activity. For example, non-V600E *BRAF *mutations tend to occur together with *KRAS *mutations [26], and inactivation of the secreted frizzled-related proteins (antagonists of WNT) by promoter methylation frequently coincides with mutations in the Adenomatous Polyposis Coli gene to achieve multi-level activation of the WNT signalling pathway in colorectal cancers [27]. Whether PIK3CA functions independently from RAS, or acts synergistically with RAS to produce additive effects on the activation of the same pathway awaits further clarification.

By extracting data from microarray, we have confirmed the up-regulation of *PIK3CA *expression in gastric cancer tissues compared with the non-neoplastic gastric mucosae and identified a large number of genes that showed a significant positive correlation in expression level with *PIK3CA*. These genes participate in diverse cellular processes with 177 as putative cell cycle-regulated genes [28] and 126 mapped to genes with known functions in cell cycle regulation, cell proliferation or DNA replication [see Additional file 2]. While some of these genes maybe induced by *PIK3CA*, others maybe co-ordinately regulated by common upstream signals. Expression data set at one point was limited in differentiating the above cause and consequence, yet it certainly revealed the complexity of the carcinogenic process and the intricate relationship of *PIK3CA *signalling with other cellular processes.

Contrary to our expectation, the incidence of *PIK3CA *mutation found in the current study (4%) is much lower compared with that observed by *Samuel et al. *(25%) [13]. The reason for discrepancy may simply be a result of sample bias as the previous study involved only a small number of gastric cancers (n = 12). However, ethnic differences can also be another possibility. The diverse pathological spectrum and aetiological factors of gastric cancers in different geographical locations may be paralleled by differences in molecular pathway of tumour development. Since our current study is only based on a Chinese population with an intermediate gastric cancer incidence, further studies involving patients from different ethnic groups will be able to address this possibility.

## Conclusion

Large-scale screening of gastric adenocarcinomas for *PIK3CA *mutations revealed a mutation incidence of 4.3%. Increased *PIK3CA *expression level was observed in gastric tumours compared with non-neoplastic mucosae. This increase in *PIK3CA *level was associated with the elevated expression of a large number of genes, which may constitute the upstream regulators or downstream targets of *PIK3CA *along the PI3K signalling pathway.

## Competing interests

The author(s) declare that they have no competing interests.

## Authors' contributions

VSWL carried out the molecular analysis, performed data analysis and drafted the manuscript. CWW, TLC, WZ assisted in the molecular analysis. KMC provided the clinical data. ASWC assisted in data analysis and edited the manuscript. SS and XC participated in the microarray study and data analysis. STY and SYL conceived of the study, participated in its design, coordination and data analysis, and edited the manuscript. All authors read and approved the final manuscript.

## Pre-publication history

The pre-publication history for this paper can be accessed here:



## Supplementary Material

Additional File 1 Primers and PCR conditions used for *PIK3CA *sequencing. Conditions for PCR and sequences of primers used in *PIK3CA *sequencing Click here for file

Additional File 2 SAM significant genes list associated with *PIK3CA *expression. SAM detected 2910 genes whose expression levels were positively associated with the *PIK3CA *expression level. Gene expression data were extracted from the microarray database containing 126 samples (90 gastric adenocarcinomas, 14 lymph node metastasis and 22 non-tumour gastric mucosae) based on a 3-fold signal above background ratio for either channel and with 80% good data [14]. Gene expression data from 20,336 cDNA clones satisfied this selection criteria and were extracted, which included a cDNA clone corresponding to *PIK3CA *(IMAGE clone number 345430, GenBank accession no. W72473). Expression value of this clone was imputed as a quantitative variable for SAM analysis. The missing values in the dataset were estimated by a K-nearest neighbours impute algorithm using 10 nearest neighbour followed by 5000 permutations in the SAM analysis. Click here for file

## Figures and Tables

**Table 1:** Spectrum of *PIK3CA *mutations in gastric adenocarcinoma

Case	Nucleotide Substitution^a^	Amino acid change	Age	Sex	MSI Status^b,c^	*KRAS *Mutation^c^	Tumour site	Tumour type	IM^d^
28	A3140G	H1047R	37	M	Stable	-	Body	Intestinal	0
100	G1633A	E545K	79	F	High	-	Antrum	Intestinal	1
240	G1624A	E542K	74	M	High	G12D	Antrum	Intestinal	1
310	A3140G	H1047R	72	F	High	G13D	Antrum	Intestinal	1

^a^Nucleotide change at the position within coding sequence, where position 1 corresponds to the first position of the start codon

^b^High, high level of MSI; Stable, microsatellite stable

^c^Analysis of *KRAS *mutations have been performed and reported previously [16]

^d^IM, the presence of intestinal metaplasia at tumour edge; 1, present; 0, absent
